# Descriptors for High Throughput in Structural Materials Development

**DOI:** 10.3390/ht8040022

**Published:** 2019-12-05

**Authors:** Matthias Steinbacher, Gabriela Alexe, Michael Baune, Ilya Bobrov, Ingmar Bösing, Brigitte Clausen, Tobias Czotscher, Jérémy Epp, Andreas Fischer, Lasse Langstädtler, Daniel Meyer, Sachin Raj Menon, Oltmann Riemer, Heike Sonnenberg, Arne Thomann, Anastasiya Toenjes, Frank Vollertsen, Nicole Wielki, Nils Ellendt

**Affiliations:** 1 Faculty of Production Engineering, University of Bremen, Badgasteiner Straße 1, 28359 Bremen, Germany; g.alexe@bimaq.de (G.A.); mbaune@uni-bremen.de (M.B.); bobrov@iwt.bremen.de (I.B.); ingmar.boesing@uni-bremen.de (I.B.); clausen@iwt.uni-bremen.de (B.C.); czotscher@bias.de (T.C.); epp@iwt-bremen.de (J.E.); andreas.fischer@bimaq.de (A.F.); langstaedtler@bime.de (L.L.); dmeyer@iwt.uni-bremen.de (D.M.); menon@iwt-bremen.de (S.R.M.); oriemer@lfm.uni-bremen.de (O.R.); sonnenberg@iwt-bremen.de (H.S.); thomann@iwt.uni-bremen.de (A.T.); toenjes@iwt.uni-bremen.de (A.T.); info-mbs@bias.de (F.V.); wielki@iwt-bremen.de (N.W.); ellendt@uni-bremen.de (N.E.); 2 Leibniz Institute for Materials Engineering - IWT, Badgasteiner Straße 3, 28359 Bremen, Germany; 3 Automation and Quality Science, Bremen Institute for Metrology, University of Bremen, Linzer Str. 13, 28359 Bremen, Germany; 4 Center for Environmental Research and Sustainable Technology, UFT, Leobener Strasse 6, 28359 Bremen, Germany; 5 BIAS-Bremer Institut für Angewandte Strahltechnik GmbH, Klagenfurter Str. 5, 28359 Bremen, Germany; 6 Bremen Institute for Mechanical Engineering (BIME), University of Bremen, Badgasteiner Straße 1, 28359 Bremen, Germany

**Keywords:** high throughput, structural materials, steel, descriptor, predictor, universal micro-hardness testing, XRD, shot peening, particle-oriented peening, speckle, DSC, dilatometry, compression, laser-induced shock wave, measuring instrument

## Abstract

The development of novel structural materials with increasing mechanical requirements is a very resource-intense process if conventional methods are used. While there are high-throughput methods for the development of functional materials, this is not the case for structural materials. Their mechanical properties are determined by their microstructure, so that increased sample volumes are needed. Furthermore, new short-time characterization techniques are required for individual samples which do not necessarily measure the desired material properties, but descriptors which can later be mapped on material properties. While universal micro-hardness testing is being commonly used, it is limited in its capability to measure sample volumes which contain a characteristic microstructure. We propose to use alternative and fast deformation techniques for spherical micro-samples in combination with classical characterization techniques such as XRD, DSC or micro magnetic methods, which deliver descriptors for the microstructural state.

## 1. Introduction

The development of new structural materials provides the basis for a large number of future-oriented applications in areas such as energy or mobility, incorporating sustainability. The fundamental question is how a specific requirement profile (e.g., required mechanical properties or corrosion resistance) can be achieved by a combination of chemical material composition and thermal, mechanical or thermomechanical adjustment of the microstructure. New approaches in materials development are required since prevailing methods are both time and resource-intensive. Today, the development of a new steel grade for engineering purposes takes typically 10 years from the very first idea to the first full industrial size melt. Next to designing the alloy composition according to the desired application, which is already highly complex due to the manifold possible combinations, the heat treatment and/or mechanical treatments for specific material properties need to be evaluated in order to present a ready-to-market product.

By introducing methods of computer aided phase diagram calculations (CALPHAD) during the end of the last century, the preliminary design of the alloy and the aspects of phase mixture could be pre-calculated for a first idea of possible material properties [1]. However, the exact determination of the mechanical properties of new types of alloys currently requires the application of numerous testing methods that differ in specimen geometry, testing time and complexity. Among the most important properties of interest are hardness and hardenability, tensile properties, impact toughness and microstructural transformations during heat treatment. Next to these, the determination of further technological characteristics such as machinability, corrosion behavior or deformability could be relevant. 

In order to overcome the limited capacities of traditional screening methods, a new method, “Farbige Zustände” [2], is being pursued in order to reduce the time needed for development of new structural materials. Based on process routes, the approach is to scan steel alloys and their processing to fulfill a given performance profile. The approach is based on a novel high-throughput method inspired by other known approaches such as thin film PVD (physical vapor deposition) sample generation for functional materials [3] or diffusion couples for structural materials [4,5,6,7]. Instead of applying classical measurement methods on macroscopic samples, the typical characterization analytics are partially transformed into methods applicable to small spherical samples (≤ 1000 µm), and some are newly developed. The approach is not to measure classical properties by refined and micro methodology but to introduce new methods measuring characteristic descriptive values comparably to hardness or stress-strain curves on spherical samples to obtain differences and trace these back to classic material properties. By doing so, the amount of stock material needed for sample generation can be reduced dramatically. Thousands of micro samples can be produced from a fraction of a kilogram of feed stock only using drop-on-demand techniques. Thereby, a maximum of flexibility in melt composition and sample size can be achieved. The applied method of high-temperature drop-on-demand techniques is most suitable combining the forming with well controlled solidification of samples to maintain the best equality and reproducibility of the initial samples produced [8].

However, the spherical samples are very challenging for most standard characterization methods, including the tensile test and thermal analysis, such as dilatometry or differential scanning calorimetry (DSC). The sub-millimeter size and the geometry strongly limit the application of classic material testing methods. Amongst classic metallographic methods, universal micro-hardness measurements and Vickers hardness testing are the only well-established methods applicable to spherical samples with diameters of 1000 µm or below. Even hardness measurements within the typical loading range of HV1 to HV10 are challenging, because of the samples being embedded in a softer embedding resin, so that the samples tend to tilt or swerve into the resin when loaded, falsifying the results. Another major drawback of these methods is the reliance of a well-prepared, polished micro cut of the samples. Therefore, the samples need to be embedded, ground and polished, making the procedure non-effective by the means of high throughput. Additionally, hardness and microstructure are a rather incomplete set of data to evaluate the potential mechanical and corrosion properties of the material. Due to this gap in methodology, alternative evaluation processes of materials capable of high throughput need to be developed.

### Content and Structure

This paper introduces a novel approach of testing spherical micro samples by means of several dedicated high-throughput methods developed within the method “Farbige Zustände.” Within the following section (Section 2) an overview on the different alternative testing methods for material characterization by the means of descriptors is given. Then, an overview on the material used and the state of delivery of the samples is given; the specific sample preparation is introduced for the different testing methods; and the heat treatment and expected material properties derived from the heat treatment are introduced within Section 3. In the following sections, the methodology of testing of the different descriptors is given and exemplary results of the descriptors obtained for the two heat treatment variants are given. A brief discussion and comparison to literature or data derived by macro material testing by entrenched non high throughput methods are given in Section 5. After the conclusion an outlook on future work is given. 

## 2. Background and Dedication of Work

The method “Farbige Zustände” deals with a set of new structural material testing methods where so-called descriptors are derived from. These descriptors are used to characterize the materials tested instead of using entrenched non high throughput measuring methods for structural materials such as the tensile test, notched bar impact test, hardness measurement, dilatometry and differential scanning calorimetry. The descriptors measured and their testing methodologies are geared towards the determination of characteristic values derived by instrumented testing procedures and specific testing equipment capable of describing intrinsic properties of specifically spherical micro samples less than 1000 µm in diameter.

Today’s measurements of material properties are mostly not based on physical quantities. For example, the classic hardness measurement is a method describing the material’s behavior under loaded penetration of a harder, geometrically well-defined body. There are various measuring methods describing the resistance of the material against elastic-plastic deformation based on this approach, such as Vickers, Brinell or Rockwell hardness. The value generated does not give any information about the intrinsic properties of the material under a real load situation in service. They are used only as a guide to likely properties in service. The advantages of the standard methods used today are their high grade of standardization and the well-defined methodology of achieving the values. Therefore, it is deemed that values measured using the entrenched methods like Vickers hardness measurements are consistent and the value can be used as guide for the expected material behavior in service. 

Accordingly, the aim of this work was to develop new methods for the measurement of material properties which are based on the approach of using specifically matched testing procedures that can be automatized, can measure in a short time and are therefore suitable for high-throughput application.

## 3. Material and Preparation

### 3.1. Intake Analysis

For the validation of the testing methods commonly employed, samples made of 52,100 bearing steel (100Cr6) were used which are available as spheres. The investigated spherical micro samples (794 µm in diameter) are produced conventionally by a roller ball production process, in which the samples are separated from a cast wire and rolled afterwards. 

The chemical composition of the spheres is given in Table 1. The amount of chromium is 0.04 ma.% lower than the limitation of the DIN EN ISO 683-17:2000-04 standard. Due to the size of the spherical particles, there is no impact of reduced hardenability because the lower chromium content is to be expected. For hardenability, increased manganese and nickel content were targeted.

**Figure 1:**
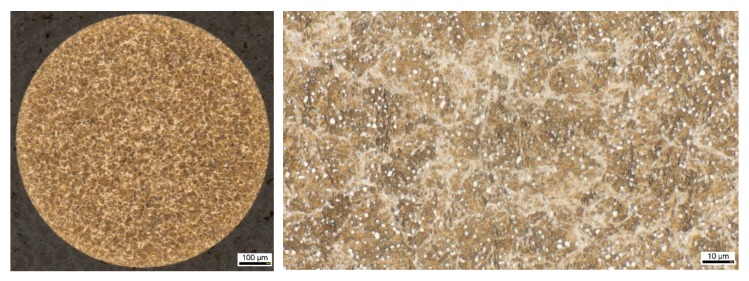
Initial microstructure of the spheres made of SAE52100 (100Cr6) (20 s, 3% alcoholic, HN0_3_).

The initial heat treatment applied to the spheres in state as delivered was a combination of quench and temper (QT) that gave a martensitic microstructure with very finely dispersed, retained austenite and spherical carbides. The microstructure was evaluated metallographically and can be seen in Figure 1. It consists of a mainly martensitic microstructure (beige/brown) with spherical carbides finely distributed (white spots). There is no light, optically visible, retained austenite present in the micro cut. These spheres then, were used for the further (heat) treatments described within the following sections.

The primary aim of the further heat treatments performed was to alter the initial microstructure with well-defined heat treatment passes giving a broad spectrum of material properties. Therefore, it first was chosen to compare the sensitivity and applicability of the descriptors by an annealed state (spheroidizing from QT state) and a hardened state with a low austenitizing temperature and a fast quenching without any further tempering of the as hardened microstructure. The heat treatment was performed using a two chamber vacuum furnace and a salt bath to prevent major oxidization and side effects from the atmosphere on the spheres.

The two microstructures aimed for were a martensitic microstructure, where a low deformability due to a combination of three major strengthening factors of metallic materials were combined via the martensitic transformation—which is a far from equilibrium reaction of iron-based, carbon containing alloys after quenching from an austenitic state, so that no diffusion was possible, and the transformation was managed by a displacive mechanism at low temperature. Therefore a series of multiple carbon, supersaturated, tetragonal, body centered crystallographic areas was generated in each austenitic grain with a high internal stress due to supersaturation with carbon and by the division of the crystallographically-differently oriented volumes being departed from each other by retained austenite. A high dislocation density is present additionally. Therefore a plasticity based on the movement of dislocations in the crystal is hampered strongly. The resulting strength and hardness is high and plastic deformation can only be reached at a rather high magnitude of stresses. Typical hardness of such structures made of 52,100 bearing steel is 800 HV1. The structure is deemed rather brittle with a high elastic deformability but has no pronounced plasticity, especially when no adjacent tempering is applied. Due to the non-equilibrium state with carbon supersaturation of martensite and retained austenite being present, thermally induced effects, including the precipitation of carbon from martensite and austenite decomposition by transformation into ferrite, are possible by heating the samples. Additionally, the transformation of martensitic microstructures into austenite can be achieved easily because of the homogeneous distribution of carbon in the martensitic crystals.

Contrarily, the microstructure of the annealed (GKZ) state consists of a homogeneous equilibrium near the ferritic matrix with a low strength due to a good plasticity of ferrite with only low hindering of dislocation movement in the crystals. The globular carbide distribution additionally results in a low deformation hampering. Therefore, the material is at a low strength with good deformability. For thermally induced transformation in the region below, an austenitic transformation at above Ac1, no significant reactions were deemed to be present, because the annealed state already gave an equilibrium near microstructure. During heating to austenitizing temperature, no transformation will happen, we assume, and the transformation into austenite is deemed to take longer due to the time needed to redistribute carbon from the globular coarse carbides.

### 3.2. Heat treatment and Results of Heat Treatment

**Figure 2:**
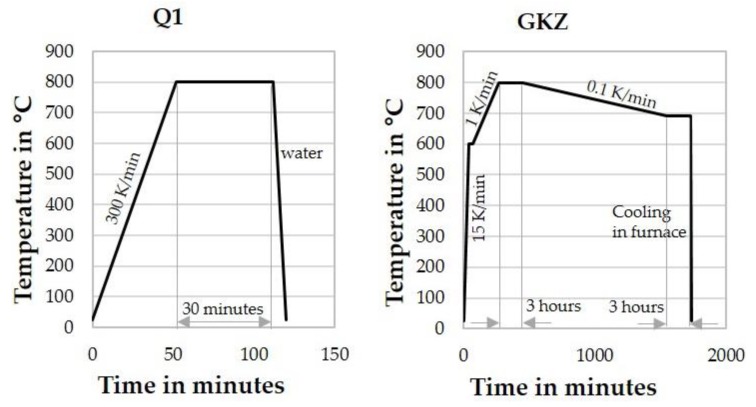
The two different heat treatments used in this study.

Two heat treatment procedures were used in this study (Figure 2). The first heat treatment (Q1) was carried out in a salt bath furnace (salt used—GS540) with a heating rate of 300 K/min from room temperature to 800 °C. This temperature was maintained for 30 min, followed by cooling using water.

The second heat treatment procedure was an annealing treatment; in particular, a spheroidizing heat treatment referred to in the following as GKZ. The samples are heated initially from room temperature to a temperature of 600 °C at a rate of 15 K/min and soaked for 30 min. This was followed by a slow heating to 800 °C (heating rate—1 K/min, soaked for 3 h). The temperature is decreased later, in a slow manner to 690 °C (cooling rate—0.1 K/min, soaked for 3 h), before cooling down to room temperature in the furnace.

These heat treatments (Q1 and GKZ) were chosen to show the applicability of the high-throughput method, where a good distinction in the various measurements could be observed. Therefore, primarily an as wide as possible range for the material’s properties was aimed for by the two heat treatments. The Q1 heat treatment results in a hard and brittle steel. The typical hardness of such structures is about 880 HV1 in macroscopic samples, which is exceptionally hard and supposedly a rather brittle state. The GKZ treatment results in a better machinable steel with a typical hardness of about 170 HV1 in macro samples. Additionally, it was expected to achieve a very good deformability due to the microstructure being very ductile.

**Figure 3:**
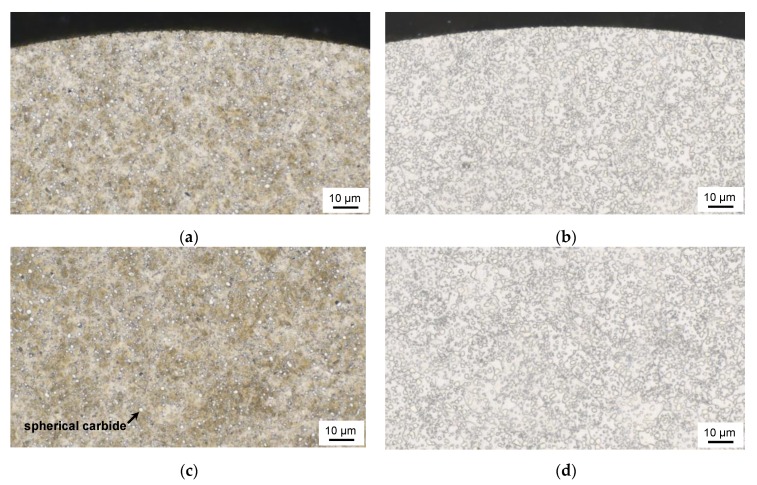
Micrographs of investigated micro samples of heat treatment conditions Q1 (**a**,**c**) and GKZ (**b**,**d**) at two magnifications.

The microstructure of the two investigated heat treatment conditions can be seen in Figure 3. The Q1 heat treatment led to very fine martensitic microstructure (beige) with no light optically visible retained austenite and evenly distributed, fine, mostly spherical carbides (white spherical areas). The microstructure is homogeneously distributed all over the micro cut, showing no segregation or unfavorable microstructure constituents. The annealing treatment led to a fully spheroidized state of cementite (white spherical areas with grey borders) within a ferritic matrix (white background). Due to the treatment, the carbide distribution is denser than in the initial state and carbide size has grown. The distribution and size are typical for such heat treatment and the very homogeneous distribution of the overall microstructure is a suitable initial state for further testing of the descriptors.

### 3.3. Preparation and Mounting

**Figure 4:**
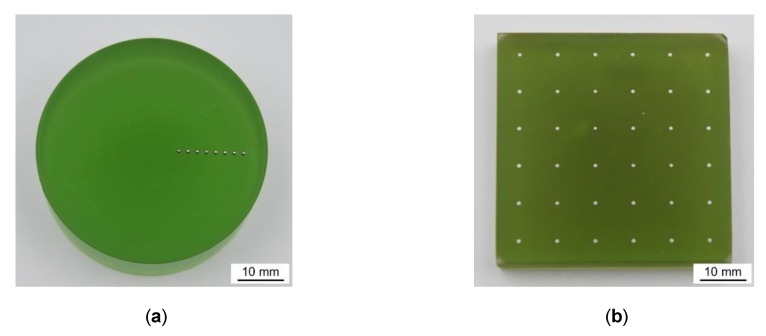
Geometry of prepared carriers: round sample carrier (**a**) and rectangular sample carrier (**b**) with embedded micro spheres.

The new testing methods proposed were developed for the investigation of micro samples. Nonetheless, not every testing setup was designed for a spherical sample geometry. For those descriptor measuring methods, which cannot work with spherical samples, the micro spheres need to be prepared by embedding in cold curing epoxy resin (Varidur 3003) and grinding to an equatorial plane. Afterwards, the surfaces of the samples are polished using diamond suspension (approximately 3 μm). In each sample carrier, several micro spheres of the same heat treatment condition are embedded with predefined positions and spacing according to the requirements of the testing procedures. There are two different sample carrier geometries used: round and rectangular (cf. Figure 4).

Table 2 assigns the particle preparation to the methods investigated in this work.

## 4. Methods and Exemplary Results

**Figure 5:**
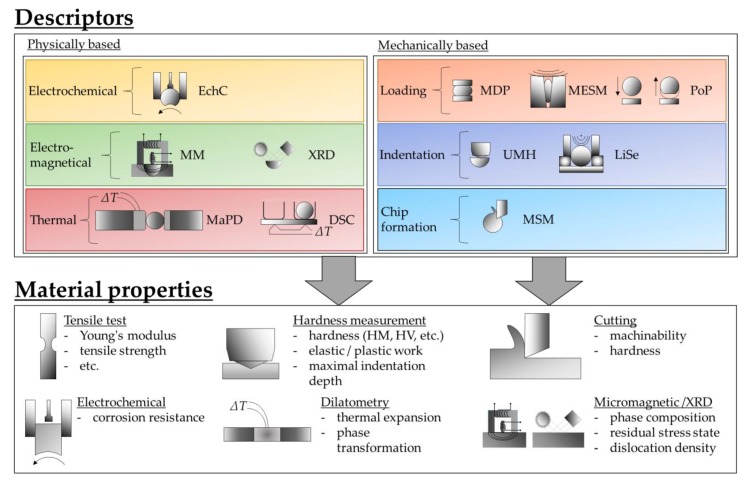
Proposed descriptor measuring methods and assumed mapping to material properties.

Figure 5 gives an overview of the descriptor measuring methods considered in this paper and their basic mechanisms. Furthermore, it schematically shows material properties (and the corresponding conventional non high throughput testing methods) to be mapped by the descriptors measured. The micro sample testing methods themselves are described in the following sections. Their mode of action is demonstrated by means of descriptors determined exemplarily for the material states GKZ and Q1.

### 4.1. Indentation Measurement by Means of Laser-Induced Shock Waves

Conventional indentation and evaluation processes usually take several seconds. One example for deducing information rapidly from adapted processes could be a novel method, which is based on laser-induced shock waves. The laser-induced shock wave indentation measurement method is referred to as LiSE hereinafter.

**Figure 6:**
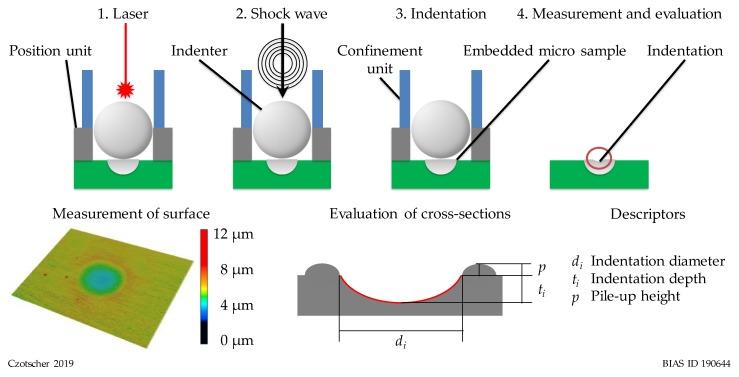
Schematic view of indentation test with confinement unit and the measurement process.

The LiSE experiments were conducted with a nanosecond pulsed TEA CO_2_ laser. The schematic set-up and measurement process are shown in Figure 6. The pulse duration of the laser beam is 100 ns with a maximal pulse energy of 6 J. Accordingly, an intensity of 1.5 GW/cm^2^ was reached with a minimal quadratic beam spot of 4 mm^2^ at a focal length of 200 mm. The spatial energy distribution of the laser beam is a top-hat with nearly uniform intensity. The high intensity laser beam creates a plasma on top of the indenter, which becomes unstable and results in a shock wave. The shock wave pushes the indenter into the test sample within microseconds. The pulse energy was set to the maximum value of 6 J during the experiments to transfer maximal pressure on the indenter ball. The shock wave impact efficiency strongly depends on the indenter diameter and the mass of the indenter [9]. The indentations were created with Al_2_O_3_ indenter balls with diameters of 3 mm. A confinement unit was placed on top of the position unit hindering the shock wave expansion to increase the acting shock wave pressure on the indenter ball. Depending on the set-up, maximum forces between 15 N and 40 N were observed.

So far, the descriptors indentation depth, indentation diameter and pile-up height were considered for correlations of material properties such as tensile strength and hardness. The descriptors were measured by means of a laser-scanning confocal microscope after the impact. Experiments revealed that the hardness can be predicted by means of neural networks when the descriptors’ depth and diameter are considered, which shows that both descriptors contain relevant information [10].

#### Results of Indentation Measurement by LiSE

**Figure 7:**
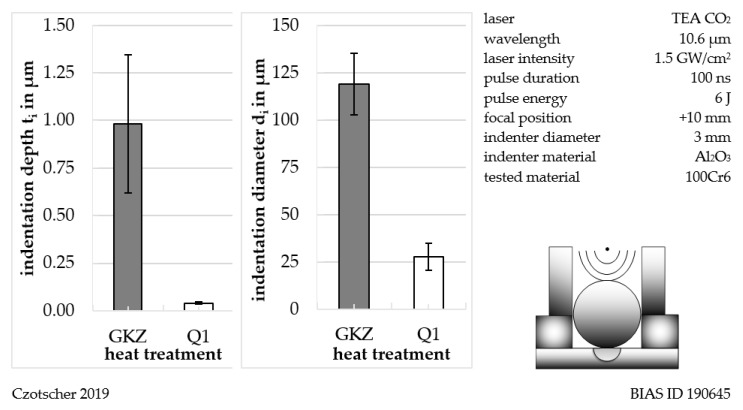
Descriptor values (indentation depth and diameter) determined by laser-induced shock wave indentation (LiSE) for the heat treatment GKZ and Q1.

LiSE descriptor values (indentation depth and indentation diameter) for the heat treatments GKZ and Q1 can be seen in Figure 7. Both the indentation diameter and the indentation depth reveal significant differences between the two heat treatments. Larger indentation depths and diameters were measured for the GKZ state. This is in agreement with the effective hardness values of the samples being around 880 HV1 for the designated Q1 heat treatment and about 175 HV1 for the GKZ treated samples. After doing a reassessment of Vickers hardness into Brinell hardness (HBW), GKZ hardness was found to be about 184 HBW.

Hardness is defined as resistance against the indentation produced by the penetration of a harder body. Thus, the decrease of hardness from Q1 to GKZ produced increased indentation diameters which can be seen for the descriptor measured as well. The physical reason is that the ferritic GKZ state with the globular carbides allows much more plasticity at lower stresses due to easy dislocation movement. The martensitic microstructure is much harder and less deformable due to an initially high dislocation density and micro stresses and multiphase design of the microstructure (martensite, retained austenite and carbides), all contributing to a reduced mobility of dislocation movement within the material. A good correlation from the testing methodological side of the descriptor-based measurement can be taken onto a standardized Brinell hardness measurement, where a hard (tungsten) ball is impinged to a flat surface producing an indentation. Compared to the standard test, the measured descriptor hardness is higher, resulting in a Brinell hardness value of 236 HBW calculated from the maximum loading (28 N) and diameter of the indenter (3 mm). The reason is possibly that compared to Brinell hardness testing, no waiting time is applied at full loading for the descriptor due to the impulsive loading of the embedded sample, which can affect the impact efficiency of the indentation process.

### 4.2. Particle-Oriented Peening Process

In order to generate information about the deformation behavior of particles due to a high velocity impact, a particle-oriented peening process is used [11]. For this purpose, single micro samples were accelerated using compressed air (maximal jet pressure p_s_ = 4 bar) to impact on a contact plate of higher hardness (hardened 100Cr6; 63 HRC), which was located at a constant distance of a = 80 mm in front of the nozzle outlet. This avoids plastic deformation of the contact plate and the particles were deformed by the impact according to their material properties (e.g., hardness or yield strength) [12].

**Figure 8:**
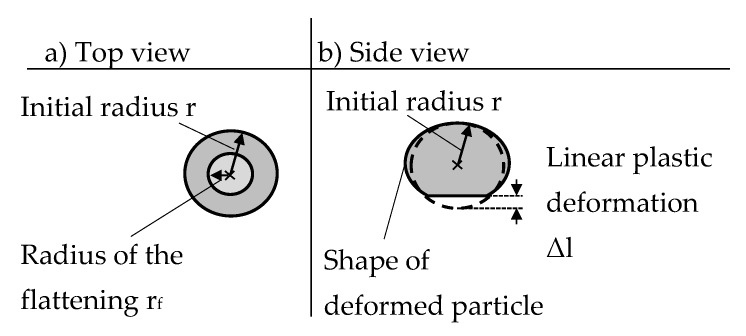
Definition of the determined radius of the flattening and the linear plastic deformation: (**a**) top view and (**b**) side view of the deformed particle.

To allow for conclusions about the material-property-determined forming-behavior of the particles they are characterized before and after the impact by measuring their dimensions. Therefore, the Zeiss SteREO.V12 microscope was used in combination with the REOObjective.435200-0000-000 objective. The descriptor “radius of the flattening r_f_” is determined by means of the light microscope after the impact (cf. Figure 8a). Utilizing the geometric relationship of the sphere segment, the initial particle radius r and the radius of the flattening are used to calculate the linear plastic deformation Δl due to the flattening (cf. Figure 8b). It is defined as difference between the initial particle radius and the distance from the center to the flattened surface after the impact (cf. Figure 8).

To further examine the influence of the material state (material composition and heat treatment), the velocities of the approaching particle (before the impact) v_1_ and the velocity of the rebounding particle (after the impact) v_2_ were determined. A system consisting of a light barrier (manufactured by “FOS Messtechnik GmbH”), two stroboscopes (“HELIO-STROB micro2” manufactured by “ELMED Messtechnik GmbH,” exposure frequency: 4 kHz) and a monochrome camera (type “DMK 5 37BUX250” manufactured by “The Imaging Source Europe GmbH”) was used for this purpose. In order be able to deduce the material dependent amount of energy that was introduced into the forming process, the deceleration of the particles by the impact ∆v_p_ was also considered.

#### Results of Particle-Oriented Peening

**Figure 9:**
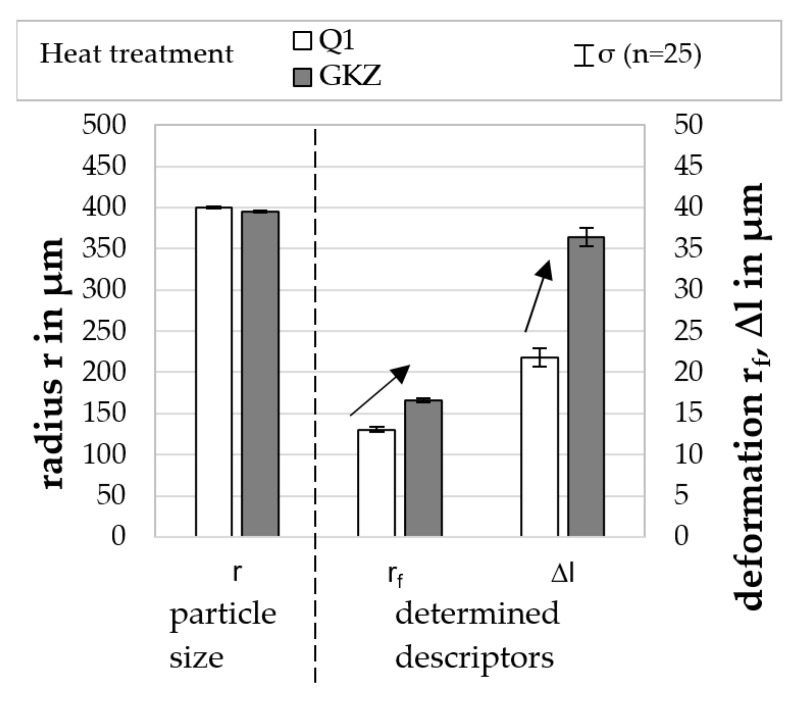
Particle dimensions determined, and descriptor values for the heat treatments GKZ and Q1 (at a jet pressure of p_s_ = 4 bar and a distance from the nozzle of a = 80 mm).

Figure 9 shows the particle sizes and the descriptors measured for the two investigated heat treatments Q1 and GKZ. It can be seen that the radii of the investigated particles are in the same order of magnitude and the impact energy, therefore, is at the same level, so that the descriptors can be compared. Both for the radius of the flattening and for the linear plastic deformation there are clear differences between the two heat treatments. For both descriptors, significantly higher values were determined for the GKZ state. The fact that, utilizing the same process parameters, the plastic deformation of these particles is higher due to their significantly lower hardness is, thus, correctly reflected by the descriptors. The high loading speed during deformation needs to be accounted for here with further comparison to, e.g., a micro compression test (see Section 4.5). The dislocation movement producing the persistent plastic deformation is limited in speed of movement. Therefore, the deformation produced at a loading speed of more than 60.000 s^−1^ is presumably producing a lower plastic deformation than the corresponding, much slower loading micro compression test 0.001 s^−1^.

**Figure 10:**
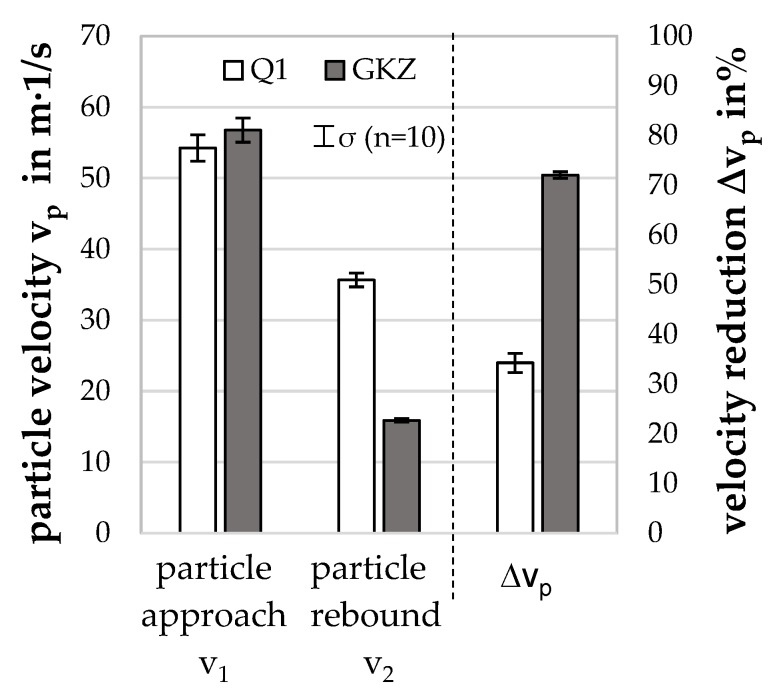
Particle velocities determined for the heat treatments GKZ and Q1 (at a jet pressure of p_s_ = 4 bar and a distance from the nozzle of a = 80 mm).

Figure 10 shows the particle velocities determined for the approaching and rebounding particles as well as their percentages in velocity reductions for the two investigated heat treatments Q1 and GKZ. It can be seen that the velocities before the impact are in the same order of magnitude, which can be explained by the matching particle sizes and the fact that no influence of the heat treatment on the particle density is expected. In contrast, the GKZ particles showed significantly lower rebound velocities than the Q1 particles. The percentage velocity reduction of 70 % for GKZ was significantly higher than for Q1, where a velocity reduction of 34 % was determined. This is well correlated with the plastic deformation of the samples expressed in the descriptor ∆l. The energy consumed during deformation of the spherical samples is bigger the lower the hardness is, and the deflected speed of the sample was, therefore, lower.

### 4.3. X-Ray Diffraction

X-ray diffraction is a method based on the interaction of X-rays with the crystal structures of the materials. The principles of X-ray diffraction analysis of steels in details are well described in [13]. For X-ray diffraction, Cr-Kα1,2 radiation was used. Micro focusing of the beam was achieved by using micro focus lens (Ø 0.2–0.3 mm). The detection and data collection were performed using a position sensitive detector. The 2θ-range was chosen to be from 60° to 163° with 0.05° steps and a 200 s collecting time instrumental broadening was taken into account by reference measurement of external LaB_6_ standard powder (NIST SRM 660a). Data analysis was performed using the TOPAS 4.2 and EVA (Bruker AXS) software. Simplified quantitative phase analysis was performed under assuming two existing phases, γ-phase (austenite) and α-phase (martensite/ferrite) for both material states. These measurement parameters made it possible to reduce the measurement and data analysis time by up to 50 min per sample.

The following data features were observed from XRD-data as descriptors: integral breadths and full widths at half maximum (FWHM) of each existing peak, their summary values divided by the number of peaks and microstructural parameters, such as crystallite size of the matrix phase. The crystallite size values were obtained from the Scherrer equation, using refined parameters. In summary—36 Q1-samples and 32 GKZ-samples were analyzed. The results represent averaged values for the given numbers of samples

#### Results of X-Ray Diffraction

**Figure 11:**
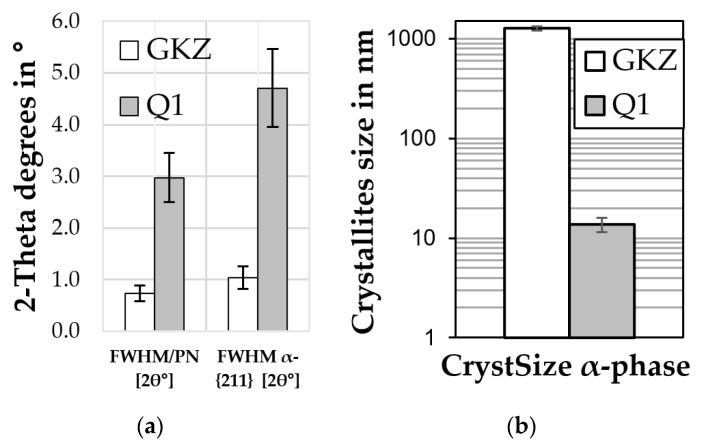
Exemplary descriptors obtained from XRD measurement results for the GKZ and Q1 states: (**a**) Summary FWHM for all peaks divided by the number of peaks and FWHM of α-{211} peak; (**b**) refined crystallite sizes for α-phase.

The results of X-ray diffraction are shown in Figure 11. As expected, the descriptors’ values are different for each of the material states [14]. The retained austenite content (and corresponding descriptors) of GKZ state was zero, as expected. The annealed state exhibiting no retained austenite in the microstructure due to the heat treatment applied. Also, the α-phase content value was 100%, due the assumption that only two phases (austenite and ferrite) existed. This, at least, is not fully correct as there was an amount of some percentage of iron carbide present, which was not taken into account during the evaluation of the data. For the Q1 variant a retained austenite content of about 5 vol.% was evaluated from the XRD measurement. This is a realistic value, as there was a low austenitizing temperature the effective solute carbon content was rather low, resulting in an increased martensite start and finish temperature, giving low retained austenite content in the final micro structure.

The evolution of peak-breadth based descriptors is seen as sensitive indicator of microstructural changes [15]. The differences between the GKZ state and the hardened state (Q1) can be easily observed: higher FWHM and IB in Q1 sample resulting in lower crystallite size and higher micro strains (refined values are 0 for GKZ state). The IB of GKZ being bigger than in Q1 state correlates well with the effective microstructure being bulk ferrite with similar orientation in GKZ against a martensitic structure with multiple crystallites in one grain. In particular, the crystallite size impacts the deformation behavior of metals, getting easier the bigger the crystallites are.

### 4.4. Micro-Magnetic Research

In general, micro-magnetic methods are based on the reaction of material with the exciting external magnetic field. The new research from this study is the high spatial resolved material characterization using micro-magnetic methods. For this, the so-called Barkhausen noise and eddy current microscope (BEMI) was used to perform measurements to characterize the microstructures of micro-scaled samples. The description and testing principles are, for example, described in [16]. Multi-frequency eddy current analysis was applied since the eddy-current loop is applied locally, directly with the sensor head at the sample surface. The multi-frequency eddy current analysis was already applied for example to characterize bainitically transformed steels [17]. In [17], it was shown that the eddy current signal correlates with the hardness of 28Cr3NiWMoVSi material, achieved by different heat treatments.

For the investigation, four exciting frequencies were predefined: 10 kHz, 100 kHz, 1 MHz and 10 MHz. Each of the signals induced in the secondary coil provided four values (imaginary and real parts (Im1–4; Re1–4), phase angle (Ph1–4) and value (Mag1–4)). This method provides 16 parameters which are defined as descriptors. The measurements were performed in the middle of the surface with manual positioning of the sensor head in the middle of the investigated surface. The measurement cycles number was chosen by 30 without removing the sensor head.

#### Results of Micro-Magnetic Analysis

**Figure 12:**
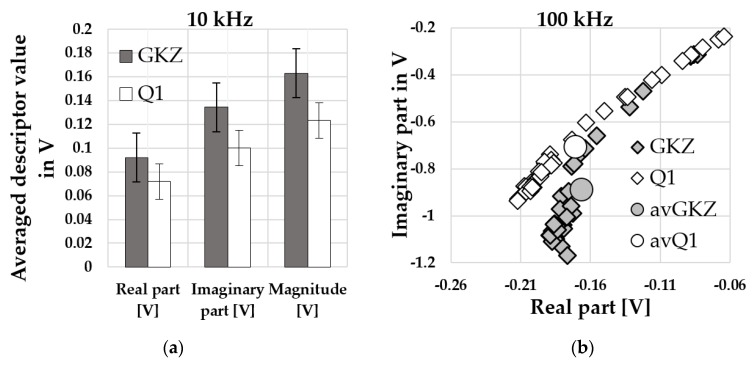
Exemplary descriptors obtained from multi-frequency eddy current analysis for the GKZ and Q1 states: (**a**) Averaged real part, imaginary part and magnitude of signal for 10 kHz; (**b**) real part value plotted versus imaginary part for all samples and averaged values measured.

Results of multi-frequency eddy current analysis for GKZ and Q1 states are shown in Figure 12. The complete results show that not all of the parameters have significant differences between the two material states. The values are small and their differences lay by the 1st, 2nd or 3rd decimal place. To compare obtained and averaged values for different material states it is necessary to consider their deviations within the investigated number of samples, which can reach up to 30%. The averaged parameters obtained with 10 kHz (Figure 12a) had the highest microstructural sensitivity without the overlapping or with small one. The relative change of the parameters decreases continuously with increasing magnetization frequency. Based on these results, a preliminary assessment of the suitability of each parameter as a microstructural descriptor can be evaluated.

On the other hand, the obtained signal is a point in the complex plane and its position is the combination of the obtained descriptors (real part/imaginary part; magnitude/phase). Figure 12b shows all the signals obtained from the 36 Q1-samples and from 32 GKZ-samples for 100 kHz. The signal points for both states are building figures which can be divided in two parts: most points are building areas separated for different states and some linear area where the signals obtained for different material states are overlapped. This kind of distribution explains also high deviations of single averaged descriptors. In Figure 12b the averaged descriptors are combined to build an averaged point on impedance plane (avQ1 for Q1 state and avGKZ for GKZ-state). The preliminary investigation showed that these deviations are dependent on sample geometry and on the positioning of the sensor head over the surface being investigated. These distributions explain also high deviations of averaged descriptor values but show the suitability of this method to qualify different material states.

### 4.5. Micro Compression Test

**Figure 13:**
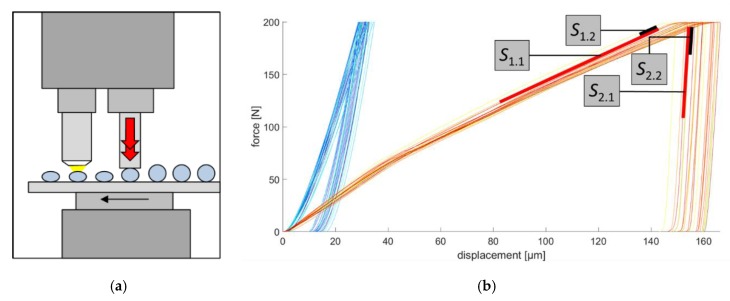
Sketch of (**a**) the micro compression test setup and (**b**) force-displacement curves derived from compression tests on spherical micro samples in the GKZ (red) and Q1 heat treatment conditions (blue) with *n* = 25 each.

To investigate the elastic-plastic deformation behavior of the spherical micro samples, a compression test was developed. For the instrumented micro compression test a hardness testing device (type ZHU2.5 of Zwick/Roell) with a maximum loading capability of 2500 N was used. A modified flat compression die enables a compressive loading of spherical and cylindrical samples (Figure 13a). The quasistatic experiments were carried out with a loading velocity of 10 N/s (force-regulated) and an unloading velocity of 0.1 mm/min (displacement-regulated). A holding time of 2 s was set when the maximum loading force was attained. The descriptors of the micro compression test on spherical samples were drawn from constantly measured force-displacement curves (Figure 13b). For the comparability of such curves, the initial sample diameter needs to be considered in the evaluation, as does the applied maximum loading force, which is usually adapted to the deformation behavior of the material [18]. However, in the following experiments the maximum loading force was set to 200 N enabling an easy and direct comparison of the curves. The characteristic values of the micro compression tests are, for example, the slope of the loading and unloading phases in the force-displacement curve, the maximum displacement *x*_max_, the plastic deformation after unloading *x*_plast_ and the mechanical work with both elastic and plastic parts.

#### Results of Micro Compression Tests

**Figure 14:**
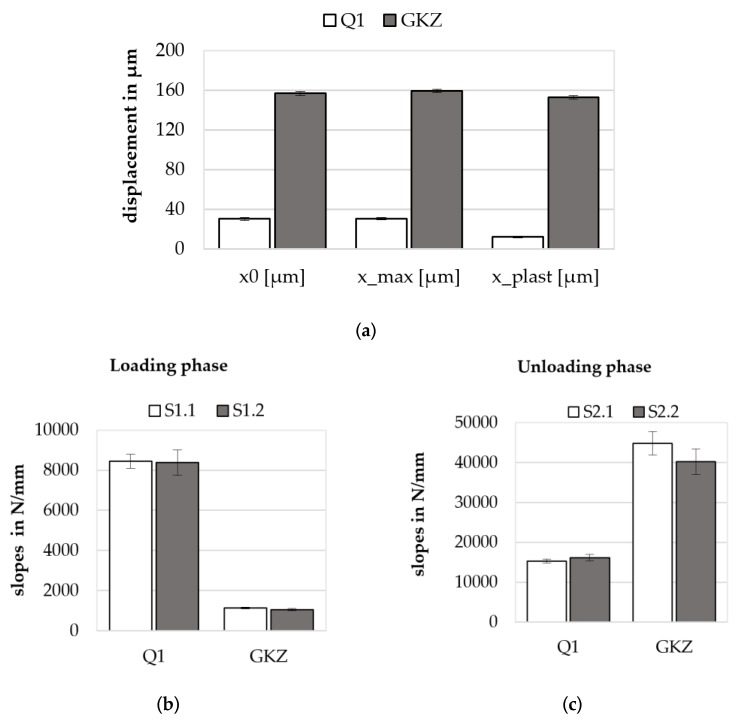
Exemplary descriptors of micro compression tests on Q1 and GKZ heat treatment conditions: (**a**) displacements; (**b**) slopes of the loading phase; (**c**) slopes of the unloading phase.

In comparison, the elastic-plastic deformation behavior of spherical samples in the GKZ and the Q1 heat treatment conditions show major differences when compressed by the same maximum force of 200 N. Force-displacement curves of GKZ (red) and Q1 (blue) (Figure 13b) clearly show significant differences, underlining that the micro compression test is a reasonable test method for comparing characteristic values of different material states. Measured descriptors of the micro compression test are shown in Figure 14. In addition to the maximum displacement *x*_max_ and the plastic deformation after unloading *x*_plast_ the displacement before holding time *x*_0_ is shown in Figure 14a. Slopes were calculated at displacements between 60% and 95% of the maximum force for the loading phase (S_1.1_) and from 95% to 60% for the unloading phase (S_2.1_), as well as for a shorter section of the curve closer to the maximum force between 95% and 96% for loading (S_1.2_) and 96% and 95% for unloading (S_2.2_) (cf. Figure 14b). Results of the slopes are presented in Figure 14b (loading phase) and Figure 14c (unloading phase).

### 4.6. Universal Microhardness Measurements

**Figure 15:**
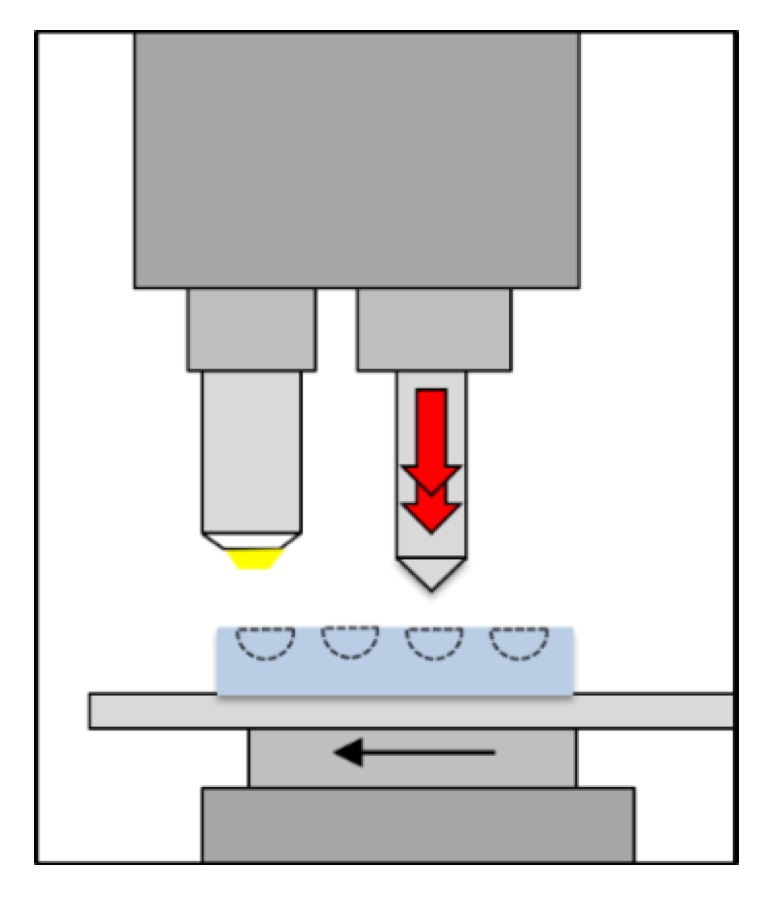
Sketch of the universal micro-hardness test setup for measurements on embedded micro spheres.

The universal micro-hardness (UMH) testing is an instrumented nano-indentation test enabling micro-hardness measurements on very small samples [19]. Figure 15 shows the sketch of the test setup. For the UMH measurements a Fischerscope H100C was used. With the Vickers indenter and the small indentation forces, very local indentation tests were carried out. In these experiments the indentation force was set to 1000 mN. The loading time, holding time at maximum force and the unloading time were set to 10 s each. As the indentations are small with diagonals of only a few micrometers the scatter of the measured values is strongly influenced by the heterogeneity of the microstructure. Therefore, 25 UMH measurements were made on each sample. The characteristic values were determined out of constantly measured force-displacement curves, taking a shape correction into account regarding the tip geometry of the indenter [20].

#### Results of Universal Micro-Hardness Measurements

**Figure 16:**
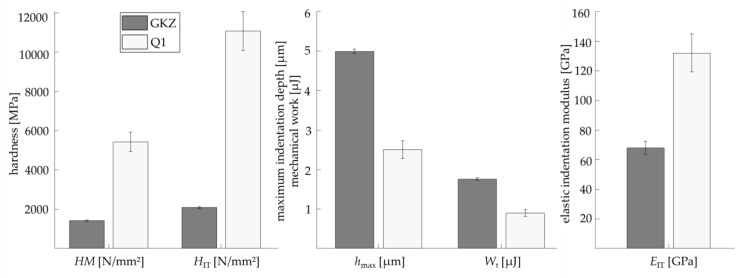
Exemplary descriptors derived from UMH measurements of the GKZ and Q1 heat treatment conditions.

Descriptors of the micro-hardness measurements are, amongst others, the maximum indentation depth *h*_max_; the Martens hardness *HM* and the indentation hardness *H*_IT_ at maximum loading; the mechanical work *W*_t_ with both elastic and plastic parts; and the elastic indentation modulus *E*_IT_, which is determined by using the tangent at the unloading part of the curve at 95% to 60% of the maximum testing force. In Figure 16 the averaged values and standard deviations of the two investigated heat treatment conditions Q1 and GKZ are shown. Significant differences between the UMH descriptors of the two investigated heat treatment conditions are obvious.

### 4.7. Micro-Extrusion Strain Measurement

Electrohydraulic incremental extrusion combined with measurements of deformation and comparison to process simulations was introduced as a tensile test equivalent in high-throughput material testing of spherical micro samples [21,22].

**Figure 17:**
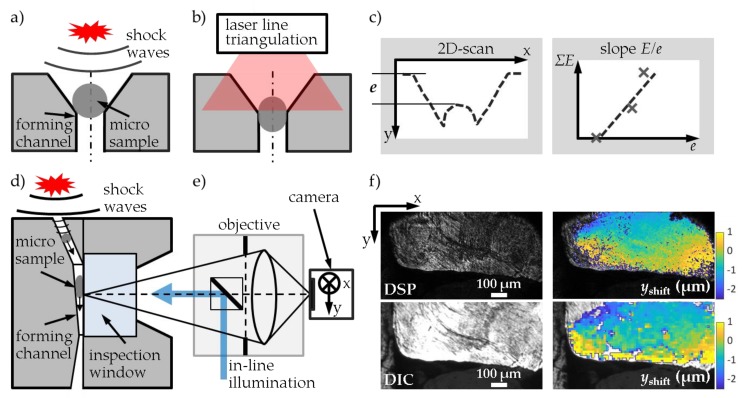
Micro-extrusion strain measurement: (**a**) forming principle for the electrohydraulic incremental extrusion and (**d**) forming through an extrusion channel with inspection window; principle of the measurement of deformations by (**b**) laser line triangulation and (**e**) speckle photography (DSP)/microscopy (DIC); (**c**) measurement of the extrusion depth *e* for the cumulated forming energy Σ*E* and (**f**) DSP and DIC images with the resulting relative displacements in the extrusion direction *y*_shift_ after a forming step.

The test procedure is illustrated in Figure 17. The electrical discharge of a current pulse generator provides the energy for the vaporization of a wire leading to plasma formation in a water filled pressure chamber. The resulting shock waves transmitting through water cause a pressure increase up to several gigapascal (GPa) within a few microseconds. The shock waves, therefore, act as an adaptable punch on the spherical micro samples (Figure 17a). Consecutive wire vaporizations transmit the forming energy incrementally to the micro sample in several forming steps. The shock wave power is set by the loading energy of the pulse generator and the vaporization energy of the wire due to material, diameter and length of the wire. The sizes of the forming steps depend on the material and dimension of the sample and of the forming channel. The shock wave is characterized by measuring the voltage and current discharge curves. The integral deformation of the micro samples is measured after each forming step by laser line triangulation (Figure 17b), in particular, the extrusion depth *e* induced by the cumulated forming energy Σ*E* (Figure 17c). The extrusion in a die along a channel with a high aspect ratio was observed in situ through an inspection window (Figure 17d) at the interface between the deformed micro sample and the sapphire window by speckle photography (digital speckle photography, DSP) and microscopy (digital image correlation, DIC) without removing the die from the extrusion system (Figure 17e). By using coherent (for DSP) and incoherent (for DIC) illumination complementarily, high-resolution speckle images and surface images were recorded between each of the consecutive forming steps along the forming path (Figure 17f). Locally resolved deformation and strain fields were calculated by means of a digital image/speckle correlation method [23,24], as shown in Figure 17f for the relative displacements in the extrusion direction *y*_shift_ after a forming step with an average shift of 13 µm. Note the relative *y*-elongation of the micro sample after forming, which results in a positive strain in the extrusion direction.

Process simulations are used to estimate strain fields and to develop a suitable forming channel design for generating tensile strain in the micro sample. According to the positive plastic strain components arising in specially designed dies, the measured strain per energy corresponds to the deformation behavior of the material tested. As a result of deformation measurements during the forming history, characteristic material values are determined, which can be used as descriptors. The specific ratio *E*/*e* is calculated as the slope of the linear fit of the cumulated forming energy Σ*E* required for the incremental increase of the extrusion depth *e* (Figure 17c). This slope was dependent on the degree of deformation *ϕ*_sd_ = ln(*d*_s_/*d*_d_) due to the die used, with the sample diameter *d*_s_ and the die diameter *d*_d_. Therefore, a normalized hardening coefficient *E*/(*e ϕ*_sd_) was calculated as a descriptor.

#### Results of Micro-Extrusion Strain Measurements

**Figure 18:**
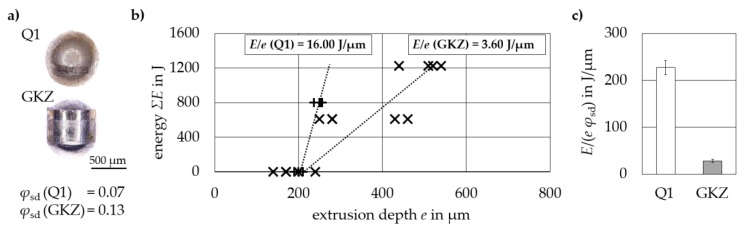
Descriptors derived for 100Cr6 samples with GKZ and Q1 heat treatment conditions: (**a**) lateral view of deformed micro samples and their respective degrees of deformation *ϕ*_sd,_ (**b**) forming energy Σ*E* as a function of the extrusion depth *e* and (**c**) normalized hardening coefficient *E*/(*e ϕ*_sd_).

Figure 18 shows the results of testing 100Cr6 samples with two different heat treatment conditions Q1 and GKZ. GKZ was extruded successfully with no cracks and Q1 displayed low formability with a tendency to crack, illustrated by the lower degree of deformation *ϕ*_sd_ achieved (Figure 18a). By stepwise increasing the energy Σ*E* (step energy *E*_st_ = 612.5 J (GKZ) and 800 J (Q1), Al99.5 wires with 0.5 mm diameter and 20 mm length) the extrusion depth *e* increased incrementally for both the Q1 and GKZ samples (Figure 18b). However, a higher forming energy was required by Q1 samples despite their lower degree of deformation. Therefore, the normalized hardening coefficient *E*/(*e ϕ*_sd_) clearly indicated the difference in ductility between the two states (Figure 18c).

A flow curve equivalent f is described by the dependency of the measured value *E*/*e* = f(*ϕ*_sd_) on the deformation degree *ϕ*_sd_. Therefore, f can be approximated by measuring *E*/*e* for micro samples at the extrusion through forming channels with different diameters leading to different *ϕ*_sd_. High-throughput testing of the flow curve equivalent is facilitated by use of a multi-tool with several forming inserts for different degrees of deformation. This multi-tool enables—due to high pressure acting over an area of 50 × 50 mm^2^—the parallel forming of several micro samples with one shock wave. In situ measurements of the local strain on the micro samples at the interface with the inspection window lead to a fast evaluation of the induced strain per energy. Hence, the flow curve’s equivalent results when testing the same sample during extrusion through a channel with a changing cross section with a higher accuracy regarding the hardening effects. The multi-tool and the in situ measurement methods are currently being tested for different materials, including 100Cr6, in order to validate their suitability to descriptor measurements.

### 4.8. Micro Sample Machining (MSM)

Machinability is a key technological feature of metallic structural materials such as steel alloys. Since material removal in chip-forming machining processes is based on local plastic deformation and shearing, the common measurands to quantify machinability (e.g., process forces, surface quality, chip form/shape, tool wear and acoustic emission) are strongly affected by the working material’s conditions [25]. The process forces in particular are directly influenced by the workpiece material’s (shear) strength and hardness, and are therefore suitable as descriptors for the characterization of the mechanical behavior of the machined material. Additionally, the machining process itself as well as the acquisition and processing of force data allow for high levels of automation and contribute to the proposed high-throughput method.

**Figure 19:**
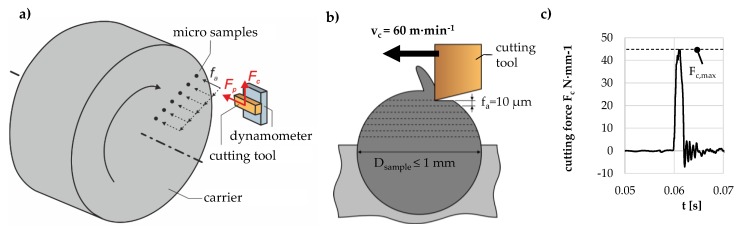
Schematic machining and measurement setup (**a**), cutting conditions for machining a single sample (**b**) and exemplary cutting force measurement for a single tool engagement (**c**).

In order to machine spherical micro samples on a conventional CNC precision lathe, multiple samples are embedded into a circular carrier in an eccentric arrangement, as described in Section 3.3 (see Figure 4). The s size requires the application of micro machining; i.e., uncut chip thicknesses of 10 µm. Machining was performed with carbide grooving tools by axial feed turning as displayed in Figure 19a, where the axial feed fa equals to the uncut chip thickness (see Figure 19b). The specimen mounting results in an intermittent, quasi-orthogonal cut with very short engagement times of the tool (Figure 19c).

Process forces were measured in cutting direction (cutting force *F*_c_) and normal to the specimen surface (thrust force *F*_p_). Since the cutting width increases with each tool engagement, an automated post-processing was implemented, dividing the measured maximum force value of each tool engagement by the effective cutting width. For both of the heat treatments investigated, experiments were performed on 25 samples. The average specific forces *F*_c_′ and *F*_p_′ and their vectorial addition *F*_z_′, and the resulting effective cutting angle *η* were evaluated for correlations to the work material properties.

#### Results of Cutting Experiments

**Figure 20:**
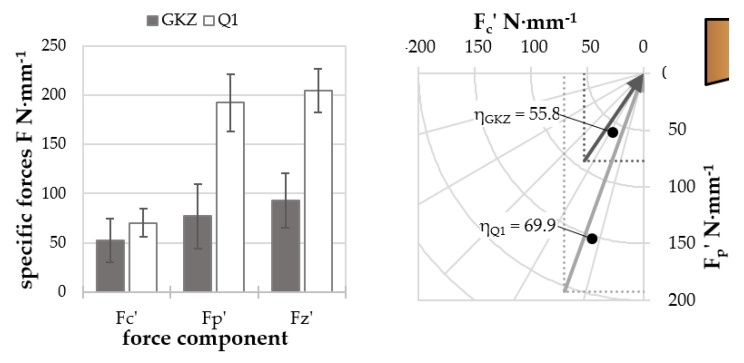
Comparison of cutting forces and effective cutting angles for the two investigated heat treatment states.

Figure 20 shows the results of cutting experiments with micro specimens of the two heat treatments GKZ and Q1, of which the former is a soft and well-machinable material and the latter a hard, brittle one that is harder to machine. The resultant vectoral addition of the process forces (F_z_′) shows a significantly higher value for the Q1 state, indicating the higher strength and hardness. For both heat treatment states, the thrust force exceeds the cutting force; however, the difference between thrust and cutting force is significantly higher for the Q1 state, which is a common phenomenon in hard machining. As a result, the effective cutting angle is much larger for the Q1 state. Therefore, the descriptors computed from cutting force measurements correctly represent the differences in the mechanical properties of the two different heat treatment states.

### 4.9. Dilatometry

**Figure 21:**
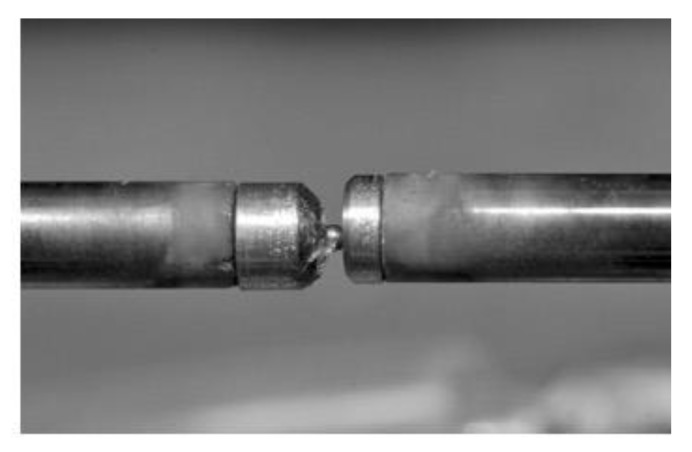
A photograph of a micro sample attached to the adaptors.

Dilatometry is a common method for characterization of phase transformation in various materials by the means of changing specific density, and therefore, changing the size and shape of a specified sample geometry. For the displacement measurements of micro samples, these are attached to molybdenum adaptor sleeves with a modified geometry (see Figure 21). The thermocouples are welded onto this adaptor for temperature measurement. These adaptors are then connected with the quartz rods of the dilatometer. The dilatometer used here was a quenching dilatometer of type 805A/D (Bähr Thermoanalyse/TA Instruments, Hüllhorst, Germany) [26,27].

#### Results of Dilatometry

**Figure 22:**
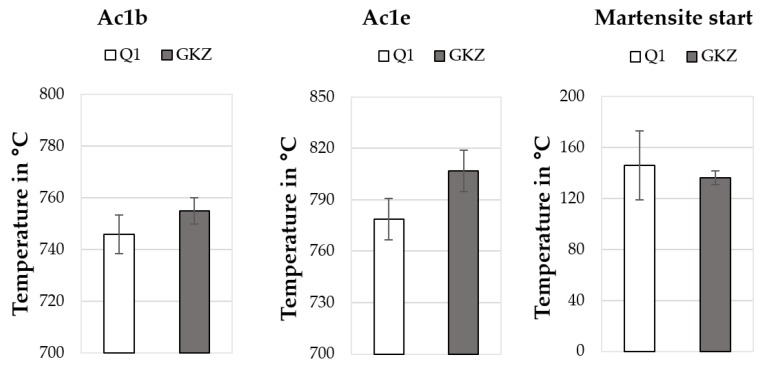
Descriptors determined from the dilatometry measurements of Q1 and GKZ heat treatment conditions.

For measuring the descriptors, both the samples were subjected to a heat treatment procedure in the dilatometer. The samples were heated to 1000 °C at a heating rate of 70 K/min and maintained at this temperature for 10 min. Before reaching 1000 °C, the sample was held at 700 °C for 3 min. This was for technical adjustment, to compensate for the expansion of the quartz rods and was below the transformation temperatures. This was followed by cooling in the dilatometer to room temperature using a nitrogen shower. The Ac1b (temperature at which transformation into austenite begins), Ac1e (end of transformation) and martensite start temperatures were determined from the dilatometer measurements (see Figure 22). Both the austenitizing temperatures (Ac1b and Ac1e) were observed to be higher for the GKZ heat treated samples, in comparison to the Q1 heat treated samples. Additionally the spread of Ac1b to Ac1e was bigger for the spheroidized state (GKZ). This is well in line with the theory and results obtained from similarly heat treated macro samples. Concerning the start of transformation into austenite, the ferritic matrix of the GKZ variant was expected to transform at higher temperatures because of the slow diffusion of carbon into the austenite being transformed from ferrite with a continuous diffusion of carbon from the globular carbides into the surroundings. The coarser the carbides are the longer the transformation will take. The transformation of a mainly martensitic microstructure (Q1) into austenite starts earlier and is accomplished faster because of the carbon being already well distributed within the matrix with no need to diffuse from a carbide into the surroundings.

However, the martensite start temperatures for the GKZ samples were slightly lower than for the Q1 samples (the higher standard deviation for the Q1 measurement needed to be taken into account). Hence, a distinction could be made for the differently heat treated samples, even though they are the same alloy.

### 4.10. Differential Scanning Calorimetry

Differential scanning calorimetry (DSC) is one of the established methods of thermal analysis. DSC is defined in DIN 51,005 as a fundamental method for the study of materials involving thermal control. In this process, there are two crucibles, one with the sample being investigated and the other with a reference material, subjected to an identical temperature program. In the case that no exothermic or endothermic reactions take place, a horizontal line is measured. If one or more exothermic or endothermic reactions take place, the heat flow curve contains peaks. The measurement of small samples with diameters of 1000 µm represents a challenge because the contact area between the sample and the crucible is very small. Furthermore, most DSC measurements are carried out with heating rates of 1 to 10 K/min. In order to meet the requirements of the high-throughput process, the DSC descriptor measurements were performed with high heating rates of up to 70 K/min and with an empty reference crucible. As descriptors, the typical characteristics of the DSC measurements such as onset, endset and peak area but also the gradient of the curve in several sections of the curve will be measured [12,26]. For the DSC measurements a calorimeter of type HT TGA/DSC 3+ (© METTLER TOLEDO, Hamburg, Germany) was used.

#### Results of Differential Scanning Calorimetry

**Figure 23:**
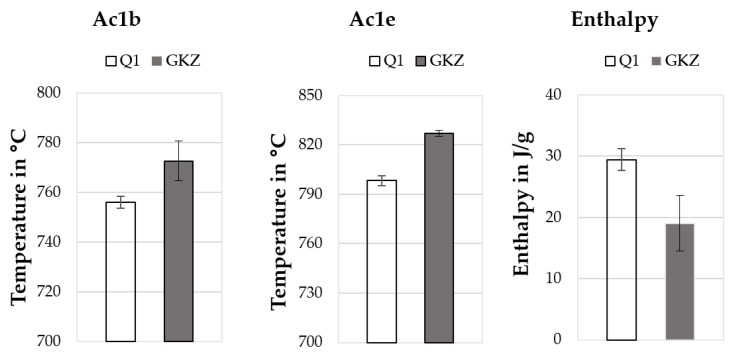
Descriptors of the austenitizing peaks derived from DSC measurements of the GKZ and Q1 heat treatment conditions.

Figure 23 shows the DSC descriptors onset, endset and enthalpy of the austenitizing peak measured with the heating rate of 50 K/min for the two different heat treatments GKZ and Q1. It can be seen that the descriptors of the both heat treatment conditions show major differences. Both the onset temperature (Ac1b) and the endset temperature (Ac1e) of the GKZ heat treated samples is higher than that of the Q1 heat treated samples. In contrast to the temperatures, the enthalpy measured on the Q1 samples is significantly higher than the enthalpy of the GKZ samples.

In addition, when heating the samples with the GKZ heat treatment, two endothermic peaks were measured (carbide formation and austenitizing). The Q1 heat treated samples have only one endothermic peak (austenitizing) when heated. It can thus be seen that the measured values of the descriptors are not the same for different heat treatments of the same alloy.

### 4.11. Electrochemical Characterization

The electrochemical behaviors of metals and alloys are directly related to their corrosion behavior and are affected by their micro structures. Thus, changes in the alloy composition, thermal treatments and mechanical treatments lead to differences in the electrochemical measurements and in the corrosion resistance of the material.

**Figure 24:**
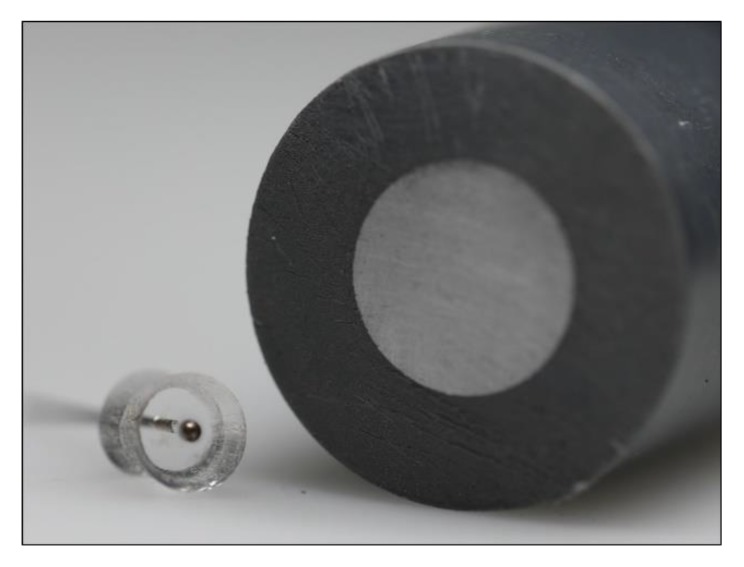
Comparison of an embedded micro and macro samples.

To investigate the corrosion behavior of metallic micro samples (700–1000 µm) the samples were embedded in PDMS and contacted by silver lacquer (Figure 24). Afterwards, the samples were investigated in a classical three-electrode cell and controlled by a potentiostat. The micro sample itself acted as the working electrode (WE); a potential was applied between the WE and a platinum counter electrode (CE), while the potential was measured via an Ag/AgCl reference electrode (RE). Three electrochemical techniques; namely, linear sweep voltammetry, cyclic voltammetry and impedance spectroscopy, were used for a comprehensive characterization. The choice of the electrolyte (pH value, chloride concentration, etc.) and process parameter can be adjusted to investigate different corrosion phenomena. Changes of the characteristic values of linear sweep voltammetry, cyclic voltammetry and impedance spectroscopy can correlate with changes in the micro structure, like phase composition and grain size, and thus mechanical properties on the different scales [28,29,30].

#### Results of Electrochemical Characterization

**Figure 25:**
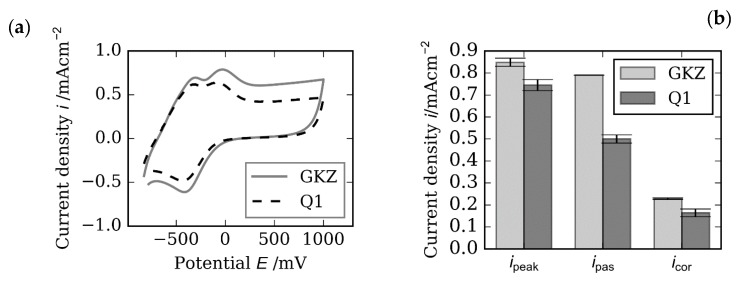
(**a**) cyclic voltammogram of GKZ and Q1 electrodes in phospahte buffer (pH 7.5); scan velocity 100 mV/s; (**b**) characteristic values derived by CV and LSV measurements.

Figure 25a shows the cyclic voltammogram of carbon steel after two different heat treatments (GKZ and Q1) in neutral phosphate buffer. Both samples led a similar course but differred in the height of the current density. Characteristic values such as the height and position of the oxidation peaks or the passive current density (*i*_pas_) provide information about the passive layer stability and the corrosion resistance. By adding sodium chloride to the electrolyte, additional characteristic values such as the pitting potential can be determined [29]. The peak heights *i*_peak_, the passive current densities *i*_pas_ derived from the CVs and the corrosion current densities *i*_corr_, derived from LSV measurements, of both samples revealed differences in the electrochemical behaviors of the steels (Figure 25b).

## 5. Discussion and Summary

Adapted and newly developed methods were presented to characterize spherical micro samples. For the actual state of the CRC1232, there are many descriptors giving similar results and using similar methods to characterize the materials. Because of most descriptors measure specific transient material responses, which for now are new and have not been analyzed in depth up to now, the variety untouched. The reason is that possible specific descriptors are in the measurement data, which could match different material properties and not only one specific value. Therefore, in the current state of affairs, all descriptors and measuring methods, despite that they might appear to be redundant, are kept and analyzed in depth in order to not lose the opportunity to learn about unforeseen possibilities to map different material properties. Additionally, all data measured is attached to the specific descriptors to allow match making in later evaluations by methods developed within the computational analysis projects.

In order to validate the concept, two heat-treated states were generated and tested. These two heat treated states differed significantly in their material properties. The GKZ state features high plasticity and low hardness because of the ferritic matrix with globular carbides embedded. Additionally, most thermally induced transformations were slower and shifted to higher temperatures in a spheroidized (GKZ) state that is rather near equilibrium condition from a Gibbs energy point of view. In contrast, the Q1 state is brittle, hard and difficult to machine due to the mainly martensitic microstructure with low retained austenite content. For thermal analysis with DSC and dilatometric methods the martensitic state is more prone to transformations happening during heating and is deemed to show the best performance by the means of a transformation into austenite due to the well distributed carbon in the microstructure.

All descriptors did feature specific differences of the measured values which could be correlated with these properties mentioned before. The LiSE hardness measurement, for example, showed a similar spread to the values obtained from the descriptor, as was expected from a comparable Brinell or Vickers hardness measurement by conventional, non high throughput methods. Especially for the GKZ state, it could be seen that the effective indentation diameter was lower than expected when the Brinell hardness was calculated from the given parameters of the descriptor and the method to obtain it. It could be seen that the very fast loading and the embedding of the samples for descriptor measurement did lead into reduced plastic deformation. Both the blackening of the sample in the resin and a decreased strain rate with increased loading speed are well in line with the theory of metal deformation. A major drawback of the LiSE method is at the moment, that the indentation can be produced very fast but the evaluation of the descriptor cannot be performed in line. In future, a direct measurement of indentation depth should be achieved, for a better automatization.

The effect of the high loading speed is present in the shot peening hardness measurement too. Therefore, a lower deformation of the actually well-deformable GKZ state is in line with the reduced plastic deformability of metals at high strain rates. The different strengths of the materials of both heat treatment states were reflected well anyway.

The magnitude of the values obtained sometimes differed from expectations (LiSE) due to specific impacts coming from the fast loading and impacts of embedding of the samples in a rather weak resin. But at least the differences could be reflected in the values and can be explained logically.

Although the mechanically based methods partly differ in whether whole or prepared spherical micro samples are examined, all the descriptive values obtained by these methods (LiSE measurement (Chapter 4.1), the particle-oriented peening (Chapter 4.2), the micro compression test (Chapter 4.5), the universal micro hardness measurement (Chapter 4.6), the micro-extrusion strain measurement (Chapter 4.7) and the micro sample machining (Chapter 4.8), were able to qualitatively represent these differences in material properties. For a discussion of the specific differences found, refer to the section of each method.

Since these methods employ different kinds of plastic deformation, due to, e.g., different process kinematics and deformation rates, it is expected that the derived measurements are sensitive to different mechanical properties, or rather, are influenced by the same mechanical properties to varying degrees. In particular, the descriptors reacted as follows to the heat-treatment-related material differences. Higher values for the GKZ state (than for the Q1 state) were found for the indentation diameter (LiSE); the indentation depth (LiSE); the flattening radius or linear plastic deformation (PoP); displacements *x*_0_, *x*_max_ and *x*_plast_ at the same test load (MDP); and the indentation depth *h*_max_ (UMH) reflecting the increased deformability of the GKZ state. Lower descriptor values for the GKZ state were found for the machining forces and the effective cutting angle (MSM) and for the normalized hardening coefficient (MESM), which reflects the better machinability of this state compared to the Q1 hardened state due to a lower resistance against plastic deformation.

Similar to the mechanically based methods, the thermo-physically and physically based methods (DSC (Chapter 4.10), dilatometry (Chapter 4.9), XRD (Chapter 4.3), micro magnetic (Chapter 4.4) and electrochemical measurement (Chapter 4.11)) allow qualitative conclusions about the state and phase composition of the materials tested. Compared to the GKZ state whose microstructure consists of spheroidized cementite in a ferritic matrix, the martensitic microstructure of the Q1 state results in a lower crystallite size and higher micro strains, which can be detected by XRD and micro magnetic analysis. The lower crystallite size of the Q1 state was deemed to be typical for a martensitically transformed microstructure due to the displacive non equilibrium character of the martensite transformation. The martensitic crystals formed multiple, thermally induced transformation products from austenitic bulk matrix grains, refining the initial structure. This also led to a higher passivation by oxidation in the given electrolyte, which was shown by the electrochemical measurement. Both the dilatometric and calorimetric measurements show a difference in the austenite start, and end temperatures for the GKZ and Q1 samples. The austenitizing temperature was higher for the spheroidized sample, as expected. The difference in measurement values of the austenitizing temperatures for the dilatometric, in comparison to the calorimetric measurements had been previously noted and was found out to be an average of 22.7 K for the micro samples [26].

## 6. Conclusions

High-throughput approaches for the development of novel materials require fast methods for sample synthesis, treatment and characterization. While characterization methods for functional materials are available for different material properties, this is not the case for structural materials. In this paper, a novel approach for short-time determination of descriptors for mechanical and physical material properties has been proposed. We presented eleven methods which can be applied to spherical micro samples with diameters of less than 1 mm, aiming at physical, chemical and mechanical quantities. The mechanical descriptors represent a wide spectrum of different stress/strain profiles and strain rates.

Based on two different heat treatments producing different microstructures on AISI52100 (100Cr6) bearing steel samples, we proved the sensitivity and applicability of these methods. All techniques presented showed characteristic differences of the values they measured that resembled the variations in microstructure, hardness and strength induced by the different heat treatments.

It can be concluded that these methods are capable of processing samples of this size and geometry and provide measurands (so-called descriptors) which allow for deducing the underlying material properties. Future work will focus on mapping descriptors and combinations of descriptors to conventional material properties over a wide range of alloy compositions and heat treatments to allow the generation of large data sets for the identification of novel materials by artificial intelligence methods.

## Figures and Tables

**Table 1:** Chemical composition of the roller balls used for the experimental work.

Material		Chemical Composition in ma.%								
		Fe	C	Cr	Mn	Ni	P	S	Si	Mo
AISI52100 (EN100Cr6)		bal	1.07 ^2^	1.31 ^1^	0.35 ^1^	0.17 ^1^	--	0.018[c] ^2^	0.35 ^3^	0.07
DIN EN ISO 683-17:2000-04	min	bal	0.93	1.35	0.25				0.15	
	max		1.05	1.60	0.45		0.025	0.015	0.35	

Note: ^1^ by atomic absorption spectrometry (AAS); ^2^ combustion analysis; ^3^ photo metric analysis.

**Table 2:** Sample preparation type for different descriptors.

Descriptor Measuring Method	Sample Preparation
X-ray diffraction (XRD)	Embedded, half sphere
Micro magnetic (MM)	Embedded, half sphere
Electrochemical characterization (EchC)	Embedded, half sphere
Laser-induced shock wave indentation (LiSE)	Embedded, half sphere
Universal microhardness measurements (UMH)	Embedded, half sphere
Micro sample machining (MSM)	Embedded, sphere
Differential scanning calorimetry (DSC)	none
Micro compression test (MDP)	none
Particle-oriented peening (PoP)	none
Micro-extrusion strain measurement (MESM)	none
Dilatometry (MaPD)	none

## Nomenclature

**Laser-induced shock wave indentation measurement method (LiSE)**	pile-up height in µm
*d*_i_	indentation diameter in µm
*t*_i_	indentation depth in µm
**Particle**-**oriented peening (PoP)**	
∆l	linear plastic deformation [μm]
∆v_p_	velocity reduction
a	distance between nozzle and contact plate [mm]
p_s_	jet pressure [bar]
r	particle radius [µm]
r_f_	radius of the flattened surface [μm]
v_1_, v_2_, v_p_	Particle velocity (_1_: approach; _2_: rebound)
σ	standard deviation [%]
**X-ray diffraction (XRD)**	
IB	Integral breadth [2θ°]
FWHM	full width at half maximum [2θ°]
CrystSize	Crystallite size [nm]
PN	Number of peaks in one diffractogram
**Micro magnetic (MM)**	
Re1, Re2, Re3, Re4	real part of eddy current signal obtained for the corresponding frequency [V]
Im1, Im2, Im3, Im4	imaginary part of eddy current signal obtained for the corresponding frequency [V]
Mag1, Mag2, Mag3, Mag4	magnitude of eddy current signal obtained for the corresponding frequency [V]
Ph1, Ph2, Ph3, Ph4	phase angle of eddy current signal obtained for the corresponding frequency [rad]
**Micro compression test (MDP)**	
*x*_max_	maximum displacement [µm]
*x*_0_	displacement before holding time [µm]
*x*_plast_	plastic deformation after unloading [µm]
*W*_t_	mechanical work [µJ]
S_2.1_, S_2.2_	slopes of the unloading phase in the force-displacement curve [N/mm]
S_1.1_, S_1.2_	slopes of the loading phase in the force-displacement curve [N/mm]
**Universal microhardness measurements (UMH)**	
*h*_max_	indentation depth at maximum testing force [μm]
HM	Martens hardness [MPa]
*H*_IT_	indentation hardness at maximum loading [MPa]
*E*_IT_	elastic indentation modulus [GPa]
**Micro-extrusion strain measurement (MESM)**	
*d*_s_	sample diameter in µm
*d*_d_	die diameter in µm
*E*_st_	step energy in J
Σ*E*	cumulated forming energy in J
*e*	extrusion depth in µm
*ϕ*_sd_	deformation degree in -
*E*/(*e ϕ*_sd_)	normalized hardening coefficient in J/µm
**Micro sample machining (MSM)**	
*f*_a_	axial feed
*F*_c_	cutting force
*F*_p_	thrust force
*F*_z_	resultant force
*η*	effective cutting angle
**Differential scanning calorimetry (DSC)**	
∆i	Enthalpy [J/g]
*T*_onset_	Onset temperature [°C]
*T*_endset_	Endset temperature [°C]
**Dilatometry (MaPD)**	
Ac1b	Temperature at which transformation to Austenite begins [°C]
Ac1e	Temperature at which transformation to Austenite ends [°C]
**Electrochemical characterization (EchC)**	
*i*_corr_	Corrosion current density
*i*_pas_	Passive current density
*i*_peak_	Peak current density
